# Gender and Social Connections as Determinants of Hypertension: A Systematic Review of Longitudinal Studies

**DOI:** 10.31083/j.rcm2511424

**Published:** 2024-11-22

**Authors:** Annalijn I. Conklin, Peter N. Guo

**Affiliations:** ^1^Faculty of Pharmaceutical Sciences, University of British Columbia, Vancouver, BC V6T 1Z3, Canada; ^2^Centre for Advancing Health Outcomes, Providence Health Care Research Institute, Vancouver, BC V6Z 2K5, Canada; ^3^Edwin S.H. Leong Centre for Healthy Aging, Faculty of Medicine, University of British Columbia, Vancouver, BC V6T 1Z3, Canada

**Keywords:** social relationships, marital transitions, risk of hypertension, aging, gender, prospective study, systematic review

## Abstract

**Background:**

Social connections impact cardiovascular diseases (CVD) morbidity and mortality, but their role in hypertension, as a CVD risk factor, and their gender inequities is less understood. This review aimed to examine the longitudinal evidence on the impact of changes in social connections on risk of hypertension among aging adults, with a specific focus on gender.

**Methods:**

A systematic search of peer-reviewed literature in Medline, Embase, Scopus, and CINAHL conducted until 10 June 2024. Prospective studies evaluating the effect of changes in living arrangement, marital status, social network, or social participation on changes in blood pressure or incident hypertension among adults aged 45 and above were included.

**Results:**

We found 20,026 records (13,381 duplicates), resulting in 6645 eligible titles/abstracts for screening and 29 texts read in full. Only six studies from three countries met inclusion criteria, with four focused on marital transitions and two on changes in living arrangement. Overall, loss of close social connections had mixed effects on changes in blood pressure or risk of hypertension. More consistent adverse CVD outcomes were observed across studies for aging adults who entered marriage or became co-living (gain of close social connections). Similarly, persistent lack of close social connections appeared to result in greater increases in blood pressure or higher risk of hypertension. Two included studies were of high quality and the rest were medium quality. Excluded studies assessing change in either CVD risk or social tie transitions were also described (n = 9).

**Conclusions:**

There is a surprising paucity of prospective evidence on social relationships as determinants of CVD risk in the aging population, despite ample research on social factors correlated with health. Limited research suggests that both gains and losses of close social connections as well as persistent lack of close social connections may alter CVD risk, but effects are specific to single-sex samples. Research and policy should prioritize causally robust high-quality studies to unravel social determinants of CVD risk as actionable evidence to inform social prescribing in CVD prevention and healthy aging strategies is still tenuous.

**The PROSPERO Registration:**

CRD42022373196, https://www.crd.york.ac.uk/prospero/display_record.php?RecordID=373196.

## 1. Introduction

Cardiovascular diseases (CVD) account for 31% of all deaths globally—more than any other cause—and heart disease is the number one killer of women in Canada [1]. CVD burden is expected to rise as a result of aging populations, with healthcare costs from hypertension alone nearly doubling from 2010 ($14B) to 2020 ($21B) in Canada [2]. Hypertension is a major biological risk factor for CVD that is also on the rise in Canada [3, 4]. Demographic shifts towards more senior populations—over 65s will represent ~25% of the total population by 2036 [5]—reinforce the urgent need for research on improving healthy aging and CVD prevention as key public health and policy priorities [6, 7]. One notable healthy aging strategy for CVD prevention is policy action to create and manage supportive environments that facilitate engagement and social connectedness among seniors [6].

Socially connected people are known to live longer [8, 9, 10], and have lower CVD morbidity and mortality in part due to improved physiological determinants of aging (e.g., obesity, hypertension) [11]. Research shows that older adults with few social relationships not only have less survivorship but the impact of social isolation is comparable with or exceeds smoking and other known risk factors of mortality [8]. A recent abstract for the American Heart Association reported that not living with a partner accounted for 10.5% of CVD mortality among US adults aged 20 to 74 years [12]. In addition, middle-aged and older adults in China whose social isolation trajectories fluctuate or remain high over time have 27% to 45% higher risks of incident CVD than those with consistently low social isolation [13]. A Cochrane review found some evidence that any social support/network intervention involving partners, informal caregivers, friends, family members, peer or lay supports delivered online or in person to individuals or groups was effective at reducing blood pressure (but not all- or cause-specific mortality and cardiovascular-related hospitalizations) [14].

Yet, the direct causal effects of alterations in social connections on developing biological risk factors of CVD among older populations is less understood [11, 15]. Higher blood pressure was found in a small genetic study of African Americans who had family-dominated social networks and fragmented networks [16]. Higher blood pressure was also reported for adults with high proportions of older children in two public housing complexes in Baltimore, US [17]. Lower odds of obesity, but not hypertension, was linked to higher numbers of health-related social connections among Latin American women living in Los Angeles, US [18]. Greater odds of hypertension in young adulthood was found for those who had become or remained social isolated since adolescence [19]. Undiagnosed and uncontrolled hypertension has been associated with network size among free-living adults aged 57 to 85 years [20]. Current literature on social isolation and CVD risk factors, however, is largely cross-sectional (i.e., subject to reverse causality) and typically combines social support with structured connections into a single index [21]; the health effects of objective lack of structured connections (e.g., social networks) are stronger than those of perceived lack of functional connections (e.g., social support, loneliness) [8, 9].

More importantly for CVD prevention and equitable care, conflicting evidence on the role of social networks or social support for CVD onset or mortality varies by gender [15, 22, 23]. Among older individuals with cardiometabolic morbidity in Canada, males were more likely to report infrequent social participation [24]. Social tie deficits associated with biomarkers of metabolic dysregulation appear stronger for US men [25]. Conversely, the protective effects of an intimate partnership for health and behaviours are more evident among men in the US [26]. Broader research showing unmarried seniors have worse health outcomes and risk factor profiles does not disaggregate data [27, 28, 29], although marital transitions may be correlated with hypertension in men but not women [30]. A sex/gender lens is absent from literature indicating that lack of social contact is linked to hypertension risk among seniors in the US [21], UK [31], and India [32]. This review therefore aimed to synthesize prospective evidence on the causal role of social relationships—a solution-linked gender variable—for risk of hypertension in aging women and men. This study is a crucial and timely opportunity to contribute new knowledge through evidence synthesis and critical appraisal to answer the empirical question, ‘*do changes in social relationships impact the risk of hypertension in aging adults and are there gender differences?*’.

## 2. Methods

### 2.1 Search and Selection

Peer-reviewed literature (articles and theses) was systematically searched using four bibliometric databases including OVID/Medline, OVID/Embase, EBSCO/CINAHL, and Scopus, until June 2024. Additionally, any potentially missed publications were retrieved through citation chaining forward and backward by reviewing the reference lists of any review articles and all included full-text articles. Any relevant full texts not available online were sought from the corresponding author, if applicable. We examined four constructs of structured social relationships: living situation, relationship status, interactions with friends/family, community group engagement [8]. Different free-text thesaurus and Medical Subject Heading (MeSH) terms were used to capture the research question concepts including: population (aging adults); exposures (changes in marital status, living arrangement, social network size, or social participation); and outcomes (changes in mean systolic blood pressure (SBP)/diastolic blood pressure (DBP) or incident hypertension). Terms used in databases are summarized in **Supplementary Table 1**. A medical information specialist performed the searches in consultation with the senior author (AIC), and developed database-specific syntaxes with no restriction on publication date. This study was registered with PROSPERO (CRD42022373196, https://www.crd.york.ac.uk/prospero/display_record.php?RecordID=373196).

### 2.2 Inclusion and Exclusion Criteria

Table 1 summarizes the review inclusion and exclusion criteria. A study was eligible for inclusion if it reported original research showing the longitudinal association between change(s) in a social tie (e.g., marital transitions, increase or decrease in social network size, etc.) with change(s) in mean SBP or DBP or with incident hypertension among middle-aged and older adults (45 years and over). Studies with either the exposure or outcome measured at one timepoint were excluded, as were studies not including data on middle- and older-age (e.g., 45 and above). In terms of trials, those studies that did not clearly focus on changes in social connections as their intervention were also excluded. Studies with cross-sectional or qualitative design, or on clinical populations were not eligible.

**Table 1.** **Inclusion/exclusion criteria based on the PECOS format**.

PECOS	Inclusion criteria	Exclusion criteria
Population	Middle-aged or older adults	Populations aged less than 45 years (i.e., late adolescents and adults in early adulthood only); clinical populations
Exposure(s)	Changes in social connection structure/structural social connections	Not evaluate changes (e.g., only baseline assessment)
Comparator	No change in/stable social connections	Missing a control/reference group of stable connection structure
Outcome(s)	Changes in mean SBP/DBP, or onset of hypertension (HR, RR or OR)	Not evaluate changes (e.g., only at one time-point)
Study design	Longitudinal studies (RCT, prospective or retrospective cohort, case-cohort, case-control)	Cross-sectional studies, qualitative studies, editorials, commentaries, and validation studies

SBP, systolic blood pressure; DBP, diastolic blood pressure; HR, hazard ratio; RR, rate ratio; OR, odds ratio; RCT, randomized controlled trial.

### 2.3 Screening

Search results were imported to Covidence Systematic Review Software for screening. Two independent reviewers (AIC, PG) screened the titles and abstracts for eligibility. Relevant full-texts were retrieved, including contacting the corresponding author, and read in full by both reviewers for final eligibility. Any disagreement between the independent reviewers was discussed and a final decision made by the senior author (AIC).

### 2.4 Data Extraction and Study Quality Assessment

Data from included studies were extracted using a standardized evidence table with pre-determined headings. Key information collected included: stated study objective, design, year, population, geographical setting, exposure description, outcome(s) measured, covariables, reported findings, author and source. Data on all results compatible with the outcome of interest were sought. Gender- and age-specific findings were included in the results field as appropriate, and any missing data or unclear data were deemed as ‘not reported’. PG performed the data extraction, and AIC verified the collected data.

The quality of included studies was assessed using an adapted checklist of itemized criteria for population-based evidence [33, 34], consisting of 28 questions and 3 response categories (‘yes’, ‘no’ and ‘cannot tell’). Criteria covered research question, design, representativeness, sampling, comparability, compatibility, completeness, results, conclusions, and generalizability. A study was deemed of high quality if around 80% of the checklist questions received a “yes” response, while a study was considered of low quality if approximately 20% of the questions received a “yes” response. Quality assessments were conducted by AIC and were used to determine confidence in the evidence. Extracted data were analyzed using narrative synthesis, with effect measures reported as mean difference, risk ratio, hazard ratio, incidence rate or odds ratio.

## 3. Results

**Fig. 1.**
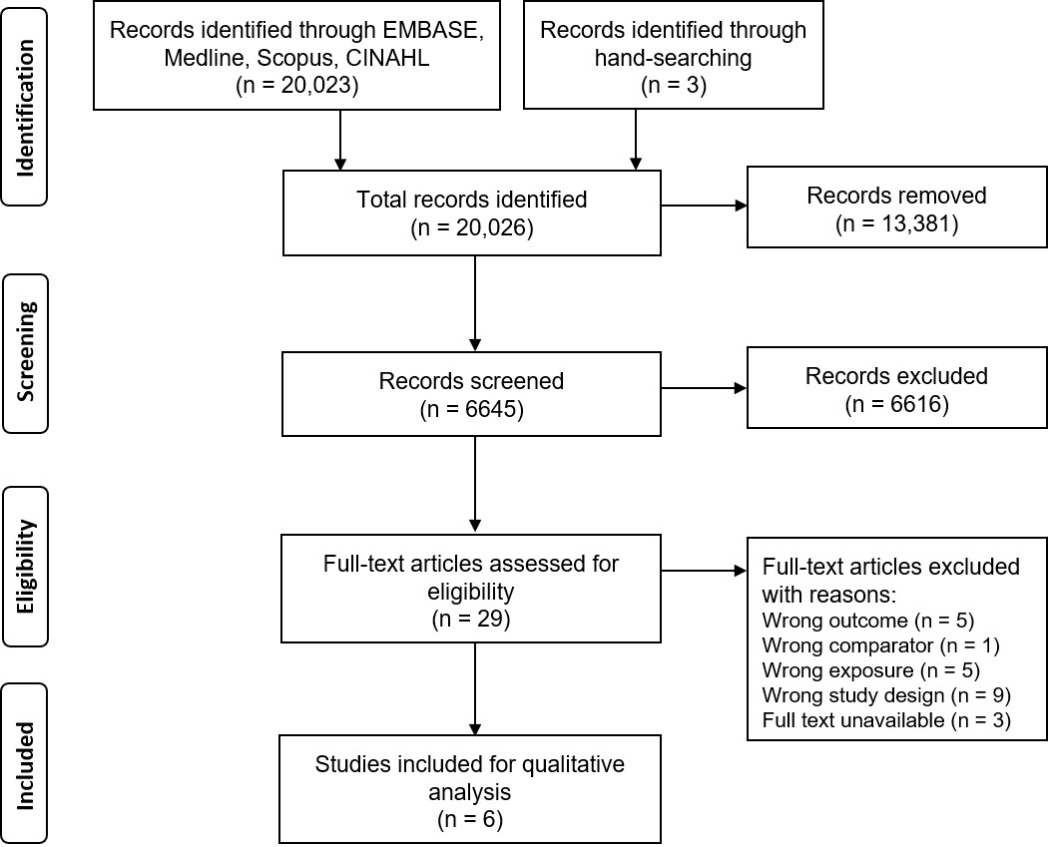
**Preferred Reporting Items for Systematic Reviews and Meta-Analyses (PRISMA) diagram of search results and study selection**.

Our searched resulted in 20,026 potential studies and, after deduplication, 6645 records were screened for eligibility; 29 eligible full-texts were read in full but only six studies met the inclusion criteria (Fig. 1). Fourteen of the 29 eligible full-texts were excluded because they were cross-sectional in design or published abstracts, and nine examined changes in either the exposure or the outcome (and not both); these nine excluded studies are also described below. Studies meeting our inclusion criteria reported work undertaken between 1999 and 2011 from Tehran, Iran [35], between 1991–1997 from China [36]; 1993–1998 from the US [37, 38]; and 2023–2024 from China [39, 40].

### 3.1 Study Quality

Four included studies were of medium quality [36, 37, 38, 39] and two were of high quality [35, 40] (**Supplementary Table 2**). Key quality concerns of included studies related to several aspects of unclear or absent elements of comparability, completeness and generalizability.

### 3.2 Study Design and Sample Characteristics

The six included studies used prospective cohort designs (Table 2, Ref. [35, 36, 37, 38, 39, 40]). One cohort included adult populations in a specific region in Iran (61% female) [35]; another cohort included young and middle-aged women in China [36]; two cohorts included postmenopausal women in the US [37, 38]; and one national cohort of older adults in China was used by two studies [39], but one included only men aged 80 and above [40]. The duration of the follow-up periods were 3 years [38], 4 years [39, 40], 5–6 years [36, 37], and nearly 10 years [35]. Sample sizes ranged from a few thousand individuals [35, 36, 39, 40] to over 35,000 individuals [37, 38].

**Table 2.** **Characteristics of included studies**.

Author/date (source)	Stated study objective	Years	Setting	Study design (data)	Study population (n)	Description of exposure	Outcome(s) measured	Covariate adjustments
Wang *et al*., 2024 [40]	To examine the link between living alone and its duration to hypertension risk in men 80 years and older	2008–2011	China (22/31 provinces)	Prospective cohort, 4 y follow-up	Older men (80+ years)	Living alone or living with family	Hazard ratio (HR) of incident hypertension	Age, education years, residence, marital status, economic independence, drinking, smoking, exercise, ADL, sleep time, no. teeth, BMI, co-morbidities, diet factors
				Chinese Longitudinal Healthy	N = 2009	Living alone time (years) before baseline:		
				Longevity Survey		Q1 = 0–6.1 years		
						Q2 = 6.1–10.6 years		
						Q3 = 10.6–19.3 years		
						Q4 = ≥19.3 years		
Wang *et al*., 2023 [39]	To investigate the correlation between living alone, alterations in living arrangements, and hypertension risk among older adults	2008–2012	China (22/31 provinces)	Prospective cohort, 4 y follow-up	Older adults (65+ years)	Living alone or living with family	HR of incident hypertension	Age, gender, education, residence, marital status, smoking, drinking, regular exercise, activities of daily living, limitations, household income, sleep time, BMI, comorbidities
				Chinese Longitudinal Healthy Longevity Survey	N = 8782	Transitions in living arrangement between wave 5 (2008) and wave 6 (2011/12) were:		
						(1) not alone/not alone		
						(2) not alone/alone		
						(3) alone/not alone		
						(4) alone/alone		
Kutob *et al*., 2017 [37]	To investigate the impact of marital transitions on health behaviors as modifiable targets for health promotion and prevention	1993–1998	USA	Prospective cohort, 5 y follow-up	Older women (50–79 years)	Two separate comparisons of changes in marital status:	Predicted change in mean blood pressure at 3 years form baseline (SBP, DBP)	Age, baseline outcome, race/ethnicity, income, education, social support, and follow-up emotional well-being, social functioning, depression score, and use of beta-blockers or antihypertensives
				Women’s Health Initiative (WHI)	N = 79,094	(1) Becoming married vs. staying unmarried as reference (single, divorced, separated, widowed)		
						(2) Becoming non-unmarried (separated, widowed) vs. staying married as reference		
Hosseinpour-Niazi *et al*., 2014 [35]	To investigate the association of marital status and marital transition with MetS and its components	1999–2011 (follow-up every 3 years)	Iran, District 13 of Tehran	Prospective cohort, 9.6 y follow-up Tehran Lipid and Glucose Study (TLGS)	Adolescents and adults (15–90 years) N = 5221 (F: 61%)	Changes in marital status had 10 possible transitions analyzed as 5 categories:	Mean percent change in MetS risk score and components (SBP, DBP)	Age, smoking status, physical activity, education levels, duration of change in marital status, BMI, medication use & history, baseline outcome
						(1) consistent single		
						(Reference for becoming married)		
						(2) consistent married		
						(Reference for exiting marriage)		
						(3) consistent divorced/ widowed		
						(4) transition to married		
						(5) transition out of marriage		
Wang 2006 [36]	To analyze the temporal relationship between earlier marital statuses and marital transitions with subsequent levels of hypertension	1991–1997	China (7 regions)	Prospective cohort, 6 y follow-up	Young and middle-aged women (20–59 years) N = 2189	Marital status categories: never married, married & marital discontinuation (divorced/separated, widowed) Marital transitions were:	Odds ratio and incidence rate of hypertension (140/90 mmHg; self-reported current treatment with medication; self-reported doctor diagnosis)	Baseline hypertension, work status, parental status, age, income, education, smoking, alcohol use, medical insurance coverage, drinking water source, cooking fuels, rural/urban location & community factors (% of residents who engage in agriculture, distance to health facility, average income and education, region)
				China Health Nutrition Survey (CHNS)				
						(1) staying married or unmarried		
						(2) changing from married to unmarried (marital dissolution)		
						(3) changing from unmarried (single, divorced/separated, widowed) to married		
Wilcox *et al*., 2003 [38]	To determine whether widowhood was associated with health and health behaviors in women	1993–1998	USA	Prospective cohort, 3 y follow-up Women’s Health Initiative (WHI)	Middle-aged & older women who are married or widowed (50–79 years) N = 38,483	Marital transitions were:	Beta-coefficient of SBP (and multiple health indicators and behaviors)	Age, education, change in income, and race/ethnicity
						(1) remained married (reference)		
						(2) married to widowed (recent ≤1 year vs. longer term >1 year)		
						(3) widowed to married		
						(4) remained widowed		

ADL, activities of daily living; BMI, body mass index; SBP, systolic blood pressure; DBP, diastolic blood pressure; MetS, Metabolic Syndrome.

### 3.3 Exposure Definition

Included studies examined transitions in marital status from baseline to follow-up, and transitions in living arrangement from baseline to follow-up or lone-living duration (Table 2). Exposure to marital transitions was defined and operationalized in different ways across studies, with all studies assessing marital dissolution (i.e., becoming divorced). One study classified marital transitions into five major categories of 10 possible changes: (1) consistent single (reference for becoming married); (2) consistent married (reference for marital dissolution); (3) consistent divorced or widowed; (4) transition to married (i.e., single to married and divorced or widowed to re-married); and, (5) transition out of marriage (married to divorced or widowed) [35]. Another study classified three categories of marital transitions: no change (staying married or staying unmarried); marital loss (change from married to unmarried); and becoming married (change from never married, divorced/separated, or widowed to married) [36]. Two different studies used subsamples of the same women’s health cohort to compare specific marital transitions. One study comparted two groups of women in separate analyses: (1) staying unmarried (divorced, widowed or never married) versus becoming married; and (2) staying married versus transition from married to divorced or separated [37]. An earlier study focused on comparing women who transitioned from married to widowed, women remaining married, women who remained widowed and women who transitions from widowed to married [38].

Exposure to transitions in living arrangement in one study in China classified participants into one of four categories based on living with family or living alone between two cohort waves [39]. Stable transitions were those who lived with family at both time points or those who lived alone at both time points; transitions out of co-living were those who lived with family at the earlier wave and then lived alone at the follow-up wave; and transitions into co-living were those who lived alone at the earlier wave but then lived with family at the follow-up wave. The other study of octogenarian men from the same cohort only examined remaining lone-living in terms of the number of years living alone prior to the beginning of the study period [40]; this duration was analyzed in quartiles.

### 3.4 Outcomes Examined

Measures of CVD risk were heterogeneous between studies. These included: a percent change in blood pressure, as a beta-coefficient or predicted mean blood pressure, hazard ratio or odds ratio and incidence rate of hypertension (Table 2). One study used a continuous risk score and all components of the Metabolic Syndrome (MetS), including SBP and DBP, for three follow-up measurements, and calculated the mean percent change in MetS and each component [35]. Another study used clinically measured blood pressure with self-reported medication or diagnosis to assess the odds and incidence of hypertension [36]. Two other studies used the same cohort to examine betas or predicted mean change in either SBP alone [38] or both SBP and DBP [37]. And finally, two other studies used the same cohort data on measured blood pressure, self-reported history of hypertension diagnosed by a physician or hypertension status self-reported by close relatives of a deceased participant, to estimate the hazard (or risk) of developing hypertension [39, 40].

### 3.5 Main Findings 

The reported findings from included studies showed both negative and positive effects on CVD risk from marital transitions over time (Table 3, Ref. [35, 36, 37, 38]) and living arrangement transitions or duration (Table 4, Ref. [39, 40]).

**Table 3.** **Summary of reported results for marital transitions**.

Quality	Author	Stable transition		Transition out of marriage	Transition into marriage	Reported results
		Remain married	Remain unmarried	Became unmarried	Became married	
High	Hosseinpour-Niazi *et al*., 2014 [35]	REF	(REF*)	M: SBP ↑ DBP ↑ W: (unemployed): SBP **↓** DBP **↓** W (employed): **SBP ↓** DBP **↓**	(*) M: SBP **↓** DBP ↑ W (not working): SBP ↑ DBP ↑ W (working): SBP ↑ DBP ↑	Changes in SBP and DBP all failed to reach significance for most marital transitions.
						Became unmarried: Males transitioning out of marriage had an increase in mean percentage of SBP (1.1%) and DBP (3.5%), relative to staying married. Females had decreases in both (range: –0.6 to –5.6%) vs staying married; only the decrease in mean percent SBP (–5.6% [95% CI: –10.7, –0.5]) was statistically significant in employed females who transitioned out of marriage status, relative to staying married.
						Became married: Males transitioning into marriage had a decrease in SBP (–0.2 mean percent change (95% CI: –2.3 to 1.8)) and an increase in DBP (2.4 (–0.7, 5.6)), relative to staying single. Females entering marriage had an increase in both SBP and DBP regardless of their employment status (range: 0.5 to 2.2), relative to staying single (REF*).
Medium	Wang 2006 [36]	REF	Odds of hypertension ↑ Incident hypertension↑	**Odds of hypertension ↑ Incident hypertension ↑**	Odds of hypertension ↑ **Incident hypertension ↑**	Remained unmarried: Women who remained unmarried had non-significant higher odds of hypertension (OR 1.64 [95% CI: 0.85, 3.17]) than women remaining married over 6 years. Women remaining unmarried had higher incidence rate for developing hypertension (IRR 1.37 [0.76, 2.45]) relative to reference.
						Became unmarried: Women who exited marriage had a significantly higher odds of hypertension (OR 2.03 [1.12, 3.70]), compared to women remaining married over 6 years. Women who transitioned from married to unmarried had significantly higher incident hypertension (IRR 1.82 [1.11, 2.99]) relative to reference.
						Became married: Women who entered marriage had higher odds of hypertension (OR 2.54 [0.98, 6.54]), compared to women staying married over 6 years. Women who became married had a significantly higher incidence rate of hypertension (IRR 2.38 [1.05, 5.35]) relative to reference.
Medium	Kutob *et al*., 2017 [37]	REF SBP **↓ DBP** ↓	(REF*) **SBP** ↓ **DBP ↓**	SBP **↓ DBP** ↓	(*) **SBP ↑ DBP ↓**	Became married: Women who entered marriage had an increase in predicted SBP (0.65 mmHg) that was significantly different (*p* = 0.027) from the decrease in women who remained unmarried (–0.29 mmHg) over 3 years. Women who entered marriage had significantly less decrease in DBP (–0.72 mmHg, *p* = 0.018) than the decline in women who remained unmarried (–1.29 mmHg) over 3 years (REF*).
						Became unmarried: Women who became divorced/separated had a greater decrease in SBP (–1.51 mmHg) than women who remained married (–0.40 mmHg), but differences over 3 years were non-significant (*p* = 0.072). Women who divorced/separated had a greater decrease in DBP (–2.45 mmHg) that was significantly different (*p* = 0.005) from the decline in women remaining married (–1.45 mmHg) over 3 years.
Medium	Wilcox *et al*., 2003 [38]	REF	**SBP ↑**	SBP **↓**	**SBP ↑**	Remained widowed: Women who remained widowed had a significant increase in SBP (0.763 mmHg, *p * < 0.01) relative to changes in women remaining married over 3 years.
						Becoming widowed: Women who became both recent and long-term widows had non-significant decreases in SBP (respectively, –0.359 mmHg and –0.709 mmHg) relative to changes in women remaining married over 3 years.
						Became married: Women who were widowed and re-married had a significantly greater increase in SBP (3.033 mmHg, *p * < 0.01) relative to changes in women remaining married over 3 years.

↓, decrease; ↑, increase; bold is statistically significant; W, women; M, men; SBP, systolic blood pressure; DBP, diastolic blood pressure; OR, odds ratio; IRR, incidence rate ratio; 95% CI, 95% confidence interval; REF, reference group for comparison; *, different reference group for comparison.

**Table 4.** **Summary of reported results for transitions in living arrangement**.

Quality	Author	Stable transition		Transition out of co-living	Transition into co-living	Reported results
		Remain co-living	Remain lone-living	Became lone-living	Became co-living	
Medium	Wang *et al*., 2023 [39]	REF	**Risk of hypertension ↑**	Risk of hypertension ≅	Risk of hypertension ≅	Remained lone-living (alone/alone): participants continuing to live alone had a higher risk of hypertension (HR = 1.24 [95% CI: 1.06, 1.45]) compared to those continuing to live with family over 4 years.
						Becoming lone-living (not alone/alone): participants transitioning from co-living had similar risk of hypertension (HR = 1.07 [0.92, 1.25]) as those continuing to live with family over 4 years.
						Became co-living (alone/not alone): participants transitioning into co-living had similar risk of hypertension (HR = 1.12 [0.94, 1.32]) as those continuing to live with family over 4 years.
High	Wang *et al*., 2024 [40]	REF	M	n/a	n/a	Duration of lone-living: higher risks of hypertension were observed among octogenarian men in the first (HR = 1.76 [95% CI: 1.16, 2.66]), second (HR = 1.56 [1.07, 2.29]) and third (HR = 1.66 [1.08, 2.55]) quartiles of living alone time, compared to counterparts living with family. There was no association for octogenarian men living alone 19.3 years or more (Q4).
			Q1: **risk of hypertension ↑**			
			Q2: **risk of hypertension ↑**			
			Q3: **risk hypertension ↑**			
			Q4: risk hypertension ≅			

≅, no change/no effect; ↓, decrease; ↑, increase; bold is statistically significant; HR, hazard ratio; 95% CI, 95% confidence interval; REF, reference group for comparison; Q1 to Q4, quartile 1 to quartile 4; M, men; n/a, not applicable.

### 3.6 Effects of Transitions out of Marriage or out of Co-living (Loss of Close Social Connections)

Three studies reported that women who transitioned out of marriage (widowed or divorced/separated) had greater decreases in blood pressure relative to changes in women remaining married [35, 37, 38]. Only employed Iranian women who exited marriage had significant declines in SBP [35], and only US women who became divorced/separated had significant declines in DBP [37]. The study including men showed transitions out of marriage were associated with greater increases in both SBP and DBP but these were non-significant [35]. Chinese women exiting marriage had a significantly higher odds and incidence rates of hypertension compared to women remaining married [36]. Finally, the risk of hypertension was similar for older adults in China transitioning from co-living to lone-living to those who continued to live with family [39].

### 3.7 Effects of Transitions into Marriage or into Co-living (Gain of Close Social Connections)

Four longitudinal studies reported greater increases in blood pressure among women who entered marriage compared to women remaining unmarried [35, 36, 37, 38]. A non-significant increase in both SBP and DBP was reported among employed and unemployed Iranian women entering marriage compared to women staying single [35]. Similarly, US women who entered marriage had significant increases in SBP compared to reference groups [37, 38], although one study showed significant declines in DBP [37]. Chinese women entering marriage had a higher odds and significantly higher incidence rate of hypertension compared to staying married [36]. One study of older Chinese adults becoming co-living found a similar risk of hypertension as those continuing to live with family over 4 years [39].

### 3.8 Effects of Remaining Unmarried or Continuous Lone-living (Persistent Lack of Close Social Connections)

Three studies examined cardiovascular effects of remaining unmarried [36, 37, 38], one study examined effects of remaining lone-living [39], and another assessed the effects of lone-living duration [40]. Women who remained widowed over time had significantly greater increases in SBP relative to women remaining married [38]. Women who remained divorced/separated had a significant decrease in SBP and in DBP; changes that differed significantly from blood pressure changes among women who entered marriage [37]. In addition, women who remained unmarried had non-significant higher odds and incidence rates of hypertension compared to women remaining married over 6 years [36]. Older adults in China who continued to live alone over four years had a 24% higher risk of hypertension compared to those continuing to live with family [39]. Moreover, octogenarian Chinese men who lived alone for up to about 19 years had significantly higher risks of hypertension, compared to counterparts living with family [40].

### 3.9 Effects of Remaining Married

One study reported declines in SBP and significant declines in DBP among women who remained married, but these decreases were less striking than the declines observed among women who became divorced/separated [37].

### 3.10 Characteristics of Excluded Studies

Of the 29 eligible articles, we found five studies examining changes in blood pressure or incident hypertension (outcome only) [41, 42, 43, 44, 45], and four studies assessing changes in social connections (exposure only) [25, 46, 47, 48]. Most of the excluded studies were done in the US [25, 42, 44, 46, 47, 48], often on specific subpopulations (median sample size of 3874), and used prospective cohort or longitudinal panel study designs with follow-up periods of two to eight years (**Supplementary Table 3**). All excluded studies focused on, or included, middle-age and/or older adults. Social tie exposures included: marital status [44, 46, 47], social isolation scale [25, 41, 42, 45], and social participation [43, 48]. One excluded study used only self-reported hypertension [48], while the others used clinically measured blood pressure.

Data from the excluded studies revealed conflicting results. Three studies examining changes in the social environment found no association with prevalent hypertension or at-risk blood pressure [25, 46, 48]. One study examined changes in marital status regarding cumulative divorces and found one or more divorces/remarriage increased the risk of heart attacks in women, while two or more divorces increased the risk in men [47]. Baseline single or widowed status was linked to greater rates of increase in blood pressure among women, while divorced status showed non-significant declines in blood pressure [44]. Other excluded studies reported that better social integration (including married) resulted in better blood recovery after acute stress [45] and lower risk of hypertension [42], especially in women [43]. By corollary, high social isolation increased the odds of new-onset hypertension in middle-aged and older adults [41].

## 4. Discussion

Heart disease is a significant problem in aging adults that has many social determinants and is not well studied in women. Aging is often characterized by adverse changes in the social environment thus loss of social relationships may explain CVD risk by increasing blood pressure and the development of hypertension. This systematic review of four medical and health sciences bibliometric databases using broad terms and no restrictions resulted in about a handful of medium-to-high quality prospective cohort studies from across the globe (Iran, US, China). The overall evidence is limited to mostly marital transition effects in predominantly female samples, with co-living transitions in both women and men or just men considered only in a single country. Overall, older women who became married and stayed consistently unmarried had adverse changes in blood pressure or increased risk of hypertension but women exiting marriage had decreased blood pressure or risk of hypertension. Newer evidence suggests lone-living may be another social determinant of hypertension for older adults in China, particularly octogenarian men. Due to the heterogeneity of cardiovascular outcomes assessed in current literature, the estimation of a single pooled effect is not yet possible to support evidence-based medicine and decision-making.

### 4.1 Findings in Context

This review gathered prospective evidence from multiple countries across diverse political and socio-cultural environments. All three countries—upper-middle to high income—have similar life expectancies of 75 and 79 years in 2022 and similar marriage rates between 5 and 8 per 1000 inhabitants (see **Supplementary Table 4**). While China and the US have similar divorce rates (2.04 and 2.4/1000) and social security coverage (95% and 97% for ages 60+), Iran has higher divorce rates (200/1000) and low social security coverage (59%). These country differences in legal, social and healthcare systems could play a role in shaping the extent to which alterations in close social ties impact individual CVD risk.

Country differences in social security and senior care systems can shape the social structures available to aging populations, particularly those who are lone-living, and thus cardiovascular health. The US has a robust social security system with pensions and Medicare that provides substantial financial and healthcare support [49], but this system faces challenges particularly with objectively isolated seniors who tend to have higher Medicare spending due to increased hospitalization and institutionalization as well as greater mortality rates. These concerns suggest that policies aimed at enhancing social connectedness among seniors could lead to both improved individual and population health and significant cost savings [50]. Traditionally senior care in China was the responsibility of families, but an aging population has increased demand for institutional care. China’s social welfare system covers five types of insurance including pensions and medical insurance [51], however disparities exist between urban and rural areas [52]. Iran is a collectivist culture which contrasts with the individualistic norms of Western societies [53], and care for seniors in Iran relies heavily on family. Iran has less comprehensive social security and healthcare services [49] which creates a care gap for seniors without strong familial connections.

Although countries globally differ in their marriage equality laws and cultural norms (e.g., cis-heteronormative or same-sex), marriage remains a social institution that creates social status meaning and an economic contract to enable access to shared resources. Unlike the US and China, Iran has a very restrictive marriage culture for women that constrains their equal right to household decision-making, seek divorce, remarry or be protected from domestic violence (**Supplementary Table 4**). Nevertheless, cross-national research has shown that the shape of marital quality trajectory among Iranian marriages was the same as US couples [53], suggesting that marriage confers common psycho-social benefits across populations despite inherent cultural differences. Another international study revealed that three marriage-specific factors contributing to marital satisfaction were common across three different cultures examined (including China) [54]. Thus, while socio-legal structures can certainly be a social determinant of health and CVD outcomes, relevant social health and family literature suggests that individual-level factors related to marriage may be stronger than broader societal-level factors. That is to say, the ideology of marriage as a recognized union between individuals with mutual support and often for the purpose of creating a family, can be considered universal.

Findings from this review indicate that specific marital transitions over time may act as unique determinants of CVD risk, with entering marriage consistently linked to worse risk profiles among aging women; effects may further depend on other social identities such as employment. Most health research on social relationships concerns the social role of being a spouse, which is consistently linked to better health, especially for men [26]. For example, partnership has been linked to improved health through reduced weight and increased healthy behaviors predominantly among men in the US [26]. Conversely, unmarried seniors have worse metabolic health status, lower survival and poorer dietary risk factors, although results are not sex-/gender-specific [27, 28, 29]. Several longitudinal studies (not meeting review criteria) showed marital transitions were not or rarely associated with prevalent hypertension [25, 35, 46, 48]. However, other longitudinal studies of baseline number of divorces [47], unmarried statuses [44], or being widowed [38] reported adverse impacts on CVD risk (change in blood pressure) and outcomes (heart attack) specifically in aging women.

Of significance for healthy aging and CVD prevention is the gendered implications of cardiovascular effects of critical life course transitions in social roles, such as entering or leaving the spousal role. As one of the most stressful life events for humans, widowhood is more common in this lives of women than men (8% versus 2% in Canada), and puts older women at higher risk of poverty and financial stress due to their traditional roles of wife and mother that limit occupational status and financial autonomy [29]. That is, transitions in social connections, particularly loss of marital bonds, will alter both the social setting and economic circumstances of older adults such that the health consequences will vary by both gender and socioeconomic status [35, 55, 56]. This review illustrated that transitions out of marriage can have heterogeneous health effects as the lived experience of marital loss through widowhood is distinct from the experience of marital discontinuation through separation/divorce [37, 38].

Both direct and indirect pathways link marital transitions and CVD risk, with health-promoting behaviors as an indirect mechanism [11]. Indeed, marriage is perhaps one of the strongest social influences on individual food choices and other health-related behaviors among older populations [57]. People who are married consume more fruits and vegetables than people who are not married, and those who have lost a partner appear to have poorer nutrition than those who have not experienced this loss [29]. The positive benefits of marriage for nutritional status also appear more pronounced for older men than women [27, 58]. Future research is needed to better understand sex/gender differences in both the direct and indirect pathways of influence between changes in close social ties and cardiovascular health.

A major finding of this review is the lack of evidence to support causal inferences about other types of social ties on the risk of hypertension. Despite the fact that a wide range of social relationships are associated with mortality and morbidity, such as living arrangement, social network, social participation, and multi-component social isolation [8, 9], this review found only two cohort studies from China on changes in living arrangement as a determinant of hypertension. Identifying this knowledge gap reveals a critical research area that must be filled to inform future evidence-based senior care. This finding was unexpected given the body of empirical research showing social isolation, including lone-living, is linked to mortality and CVD outcomes [59, 60]. The literature on social isolation and hypertension continues to be limited by the use of a single index that combines structural (marital status) and functional (marital support) components into a single index; some of the review’s excluded studies with repeated measures [21, 42, 43, 45] also shared this methodological concern with previous cross-sectional research on seniors [21, 31, 32]. Unpacking different types of social relationships and their alterations is necessary to reveal intervention targets and personalize hypertension and CVD prevention care for older adults. Social relationships are a gender-related solution-oriented factor that could provide important health-promoting benefits to aging adults in terms of lowering CVD risk, and therefore deserves more attention in research. There is a clear need for more robust investigations in aging women and men to determine which changes in social relationships truly impact blood pressure or hypertension and alter poor CVD outcomes—a question this review cannot yet answer.

### 4.2 Implications of Results for Practice, Policy and Future Research

There are some implications of this review for clinical practice and research. Elucidating the effects of changes in marital status on systolic blood pressure in aging adults, particularly aging women, can help healthcare professionals identify potential risk factors for subclinical CVD and better personalize care to specific subpopulations. The evidence for a possible causal effect of close social connections on blood pressure highlights the importance of interprofessional collaboration, suggesting that health professionals, counselors (e.g., clinical social workers), and support groups should be integrated to help patients navigate these social life changes with a focus on both mental and physiological well-being. Since this review found most included studies used sex-specific samples, future research needs to give greater attention to sex/gender differences through disaggregated data from within the same population. Despite ongoing calls to advance women’s (heart) health through sex/gender-based research [61, 62], current European and American Clinical Practice Guidelines (CPGs) in cardiology do not sufficiently account for gender-specific medicine or gender equality of the social determinants of health [63]. From a sex/gender perspective, future CVD prevention research on social relationships may need to consider female hormonal changes or supplementation in later life, pregnancy history, and (peri-) menopause status that are all female biological factors that could mediate, moderate or confound the effects of transitions in close social relationships on hypertension and other CVD risk factors. Finally, future research on this important topic that incorporates a sex/gender lens may also need to account for the mediating or moderating role of changing stress levels, social support networks, or lifestyle habits. Great scope exists to shed more light on this complex interplay between the social dynamics of aging and changes in blood pressure or risk of hypertension among both women and men.

### 4.3 Strengths of the Review Methodology

There are many strengths of this review, despite the limited literature that exists on alterations in social relationships and the risk of hypertension. This is the most comprehensive to date, with searches conducted by two independent reviewers in four databases covering literature from multiple medical and health-related disciplines. In particular, this review adds valuable insights on the current evidence base as it explicitly searched a range of terms for four different types of structured social connections and not simply one aspect of this multidimensional construct. The review placed no limits on publication date to allow for potentially older studies. It used broad terms in order to ensure the widest possible literature was captured. Finally, and most importantly, this review helps to advance knowledge on women’s heart health given that women are an under-studied population in cardiovascular research [1]. This review incorporated a gender lens in the research question, data extraction, and interpretation, and gave explicit attention to possible differences in the reported results for women and men when included studies reported disaggregated data.

### 4.4 Limitations of the Review Methodology

Since this systematic review predominantly focused on longitudinal studies in published literature, the search likely missed other prospective evidence particularly from grey literature not included in the databases searched; indeed, one dissertation was found through hand-searching. Our eligibility criteria restricted results to studies where both the exposure (social relationships) and outcome (a cardiovascular risk factor) were measured at multiple timepoints, and thus may have been too strict in reducing the number of articles included in the review. In addition, our search terms focused on subclinical CVD, namely change(s) in mean SBP or DBP or incident hypertension and therefore likely missed relevant longitudinal studies that measured multiple health indicators or focused on other CVD-related outcomes, such as the two hand-searched studies included in the review.

## 5. Conclusions

This comprehensive systematic review addresses healthy aging concerns and the rising problem of CVD burden by examining changes in the social environment and possible gender disparities. Despite a large literature on social relationships and health, robust population-based prospective evidence on social relationships as (causal) determinants of CVD risk is still scarce. Only a small body of research has developed on the alterations of close social connections, namely marital transitions and co-living changes, that reflect the dynamic period of social life changes in aging adults. It was clear that persistent lack of close social connections as well as gains and losses of close social connections had an impact on CVD risk. However, the direction and size of impact differed for each sex-specific sample, for the type of transition and for the intersection of gender and employment. Thus, firm conclusions about the social context as a determinant of hypertension in aging women and men are difficult to draw.

If public health and policy aim to promote healthy aging and prevent CVD burden through social engagement, then more robust research is needed to reproduce and add actionable evidence to this burgeoning literature. Future investigations need to measure change in both the exposure and the outcome with temporal separation, reproduce results disaggregated by sex/gender within the same population, and examine whether and for whom reductions in different types of social relationships increase blood pressure or the risk of hypertension in aging adults.

## Availability of Data and Materials

All relevant data are publicly available in the published literature.

## Supplementary Material

Supplementary material associated with this article can be found, in the online version, at https://doi.org/10.31083/j.rcm2511424.
