# Supplementary material for: Gender and Social Connections as Determinants of Hypertension: A Systematic Review of Longitudinal Studies
Source: Rev Cardiovasc Med. 2024 Nov 22;25(11):424. doi: 10.31083/j.rcm2511424 (PMC11607512; doi:10.31083/j.rcm2511424)
Supplement: Supplementary file 1 [file 2153-8174-25-11-424-s1.zip › Supplementary Material-V2.docx]

**Supplemental Table 1. Example of search terms used in Ovid/Medline.**

| **#** | **Query** |
| --- | --- |
| 1 | Aging/ or Aged/ or exp "Aged, 80 and over"/ or Frail Elderly/ or Middle Aged/ or Geriatrics/ or Retirement/ or age factors/ or "age of onset"/ |
| 2 | (middle age* or elder* or senior* or aging or ageing or geriatr* or senescen* or retire* or sexagenarian* or septuagenarian* or octogenarian* or nonagenarian* or centenarian* or supercentenarian* or old* adult* or old* person or old* people or over 65 or age of onset or age factor*).ti,ab,kf. |
| 3 | 1 or 2 |
| 4 | Single Person/ or exp Residence Characteristics/ |
| 5 | (single person* or single people or single individual* or liv* alone or living arrangement* or single?ness or co?living or lone?living or living situation* or lone?dweller*).ti,ab,kf. |
| 6 | Marital Status/ or Divorce/ or Marriage/ or Widowhood/ or Spouses/ |
| 7 | (marri* or marital* or divorce* or widow* or spous*).ti,ab,kf. |
| 8 | Community Participation/ or Social Participation/ or Interpersonal Relations/ or Social Integration/ or Social Interaction/ or Social Inclusion/ or Social Support/ or Social Networking/ or Peer Group/ or Social Environment/ or Social Factors/ or "Social Determinants of Health"/ or Social Identification/ or Social Vulnerability/ or Social Isolation/ or Social Alienation/ |
| 9 | ((social* or communit* or inter?person* or peer*) adj3 (alienat* or bond* or participa* or relation* or integrat* or interact* or connect* or activit* or engag* or involv* or inclus* or exclus* or circumstanc* or isolat* or seclu* or support* or network* or environment* or group* or contact*)).ti,ab,kf. |
| 10 | or/4-9 |
| 11 | Hypertension/ or essential hypertension/ or "hypertension, malignant"/ |
| 12 | (hypertensi* or HBP or BP or SBP or DBP or blood pressure or systolic pressure or pulse pressure or diastolic pressure).ti,ab,kf. |
| 13 | 11 or 12 |
| 14 | risk/ or risk assessment/ or risk factors/ or heart disease risk factors/ or cardiometabolic risk factors/ or morbidity/ or prevalence/ or Incidence/ |
| 15 | (risk* or incidence* or preval* or morbid*).ti,ab,kf. |
| 16 | 14 or 15 |
| 17 | Case-Control Studies/ or Longitudinal Studies/ or Prospective Studies/ or Cohort Studies/ or exp Randomized controlled trial/ or Retrospective Studies/ or Follow-Up Studies/ |
| 18 | (longitudinal or prospective or retrospective or cohort or case?cohort or trial* or randomi?ed or case?control or observational stud* or experimental stud* or randomi?ed controlled trial* or RCT).ti,ab,kf. |
| 19 | 17 or 18 |
| 20 | 3 and 10 and 13 |
| 21 | 3 and 10 and 13 and 16 |
| 22 | 3 and 10 and 13 and 16 and 19 |
| 23 | (Social relationships and physiological determinants of longevity across the human life span).m_titl. |
| 24 | (Social relationships and hypertension in late life: evidence from a nationally representative longitudinal study of older adults).m_titl. |
| 25 | (Social participation and the onset of hypertension among the middle-aged and older population: Evidence from the China Health and Retirement Longitudinal Study).m_titl. |
| 26 | 23 or 24 or 25 |
| 27 | 22 and 26 |

**Supplemental Table 2. Quality assessment of the included study.**

| **Quality Criteria** | **Kutob et al. (2017)** | **Hosseinpour et al. (2014)** | **Wang et al 2024** | **Wang et al 2023** | **Wang 2006** | **Wilcox et al 2003** |  |
| --- | --- | --- | --- | --- | --- | --- | --- |
| **Question:** Does the publication address a clearly focused issue? (i.e. clear statement of research questions & objectives) | Y | Y | Y | Y | Y | Y |  |
| **Design**: Was study design described? | Y | Y | Y | Y | Y | Y |  |
| **Design:** Was the method chosen & were data sources appropriate to the research question/study objective? | Y | Y | Y | Y | Y | Y |  |
| **Design:** Was data collection & analysis described? | Y | Y | Y | Y | Y | Y |  |
| **Design**: Demonstration that outcome of interest was not present at start of study | U | U | Y | Y | Y | Y |  |
| **Design:** Was exposure of interest defined and ascertainment operationalized? | Y | Y | Y | Y | Y | Y |  |
| **Design:** Was a control group used to compare outcomes? | Y | Y | Y | Y | Y | Y |  |
| **Representativeness:** Was group exposed representative of the general population (i.e. not based on convenience sample, occupation-specific, single centre, etc.)? | N | Y | Y | Y | Y | N |  |
| **Representativeness:** Were those not exposed also drawn from the same community (vs. a different source)? | Y | Y | Y | Y | Y | Y |  |
| **Sampling:** Was the sampling strategy clearly defined & justified | Y | Y | Y | U | N | Y |  |
| **Comparability:** Did the study control for bias (e.g. measurement bias, selection bias, social desirability bias, etc)? | Y | Y | Y | Y | Y | U |  |
| **Comparability:** Were factors possibly related to both exposure and outcome identified? | N | U | Y | Y | N | N |  |
| **Comparability**: Did the study adjust for key confounders as a covariate, including baseline outcome (if relevant)? | Y | N | Y | N | N | N |  |
| **Comparability:** Were groups comparable at baseline? | N | Y | N | N | U | N |  |
| **Comparability:** Did the study perform subgroup analysis? (i.e. sex, age, socioeconomic status (SES), …) | N | Y | Y | Y | N | N |  |
| **Completeness:** Was follow-up long enough for study objectives? | Y | Y | Y | U | Y | U |  |
| **Completeness:** Could all likely effects have appeared in the study’s timescale? | U | Y | Y | Y | Y | U |  |
| **Completeness:** Could the effect be lasting/ Not transitory? | N | Y | Y | N | Y | N |  |
| **Completeness:** Was follow-up sufficiently complete (ideally, >80% participants accounted for)? | Y | N | Y | N | U | U |  |
| **Results:** Were main findings reported & do they address the research question? | Y | Y | Y | Y | Y | Y |  |
| **Results:** Was the choice of statistical analysis appropriate? | Y | Y | Y | Y | Y | Y |  |
| **Results:** Was the primary outcome measure valid and reliable? | Y | Y | U | U | Y | Y |  |
| **Results:** Were tables/ graphs usefully labelled/ understandable? | Y | Y | Y | Y | Y | Y |  |
| **Conclusions:** Were results compared with those of other studies, even if contradictory? | Y | Y | Y | Y | Y | U |  |
| **Conclusions:** Is the interpretation appropriately based on results & alternative explanations explored? | Y | Y | Y | Y | U | U |  |
| **Conclusions:** Do the findings support the conclusions? | Y | Y | Y | Y | Y | U |  |
| **Generalizability:** Can results be applied to other settings? | N | N | N | N | U | N |  |
| **Generalizability:** Were all important outcomes/ results considered? | Y | Y | N | Y | N | N |  |
| **Score (out of 28)** | 20 | 23 | 24 | 20 | 19 | 13 |  |
| **Quality criteria met (%)** | **71%** | **82%** | **86%** | **71%** | **68%** | **46%** |  |
| **Overall quality level** | **M** | **H** | **H** | **M** | **M** | **M** |  |
| *Adapted from the Effective Public Health Practice Project (EPHPP) and Newcastle-Ottawa Scale (NOS). Y, yes; N, no; U, unknown; H, high; M, medium; L, low. high: >=80% yes; medium: 20-80% yes; low <20% yes. | | | | | | | |

**Supplementary Table 3. Summary of excluded studies.**

| **Author / date** | **Stated study objective** | **Setting, years** | **Study design** | **Study size & population** | **Description of exposure** | **Outcome(s) measured** | **Reported effects on blood pressure or hypertension** | **Covariate adjustments** |
| --- | --- | --- | --- | --- | --- | --- | --- | --- |
| ***Changes in outcome only (Hypertension Onset)*** | | | | | | | | |
| Grant et al., 2009 | 1. To assess the  relationship between social isolation and acute cardiovascular reactivity and recovery.  2. To investigate associations between social isolation and  lipid responses to acute stress  3.To investigate the  relationship between CAR, cortisol diurnal slope, and  cortisol output over the day and social isolation | London, England  (1985-88) | Experimental | Working adults  (45-59 years) free of hypertension or medications  (n = 238) | Low, medium, and high social isolation groups based on an index (living alone, below monthly contact with family, no contact with friends). | Difference between baseline SBP and post-task SBP (recovery) | Return to normal SBP from baseline after a stress test was better in low social isolation groups: mean difference averaged 4.50 (SD 10.1), 7.64 (SD 9.2), and 7.47 (SD 8.4) mmHg in the low, medium, and high social isolation groups.  DBP recovery did not differ between social isolation groups. | age, employment, BMI, smoking status, and baseline loneliness. |
| Gallo et al., 2003 | To compared cardiovascular risk profiles of married or cohabitating women with high levels of marital satisfaction with those of women with moderate or low satisfaction and with those of women with no partner at two time points | United States  (1983-87) | Prospective Cohort | Middle-aged women free of risk factors (42–50 years)  (n = 493) | Marital status and marital satisfaction | Levels and trajectories of blood pressure | Relative to the satisfied group, single women exhibited a significant rate of increase in SBP (slope of 0.43) and in DBP (slope 0.34). Widowed women had a significant increase in DBP (slope 0.50), but the SBP increase was non-significant.  By contrast, divorced women had a decrease in SBP but no change in DBP. | Menopausal status, hormone replacement therapy, medication use, proportion of visits with HRT, ethnicity. |
| Yang et al., 2015 | To examine the prospective associations between social integration and social support and change in systolic blood pressure (SBP) and hypertension risk over time | United States  (2005-11) | Longitudinal  National Social Life, Health, and Aging Project | Older adults (57 to 85 years)  (n= 1264) | Social integration scale (marital status, contact with family or friends or neighbors, participation at church, meetings or volunteering) | Change in logSBP and predicted probability of hypertension | No effect of social integration on change in logSBP.  Compared with the most socially integrated, those with the lowest level of integration at Wave 1 had 75% greater risk of hypertension (95% CI: 1.04, 2.95) at wave 2. | Age, sex, race, education, antihypertensive medication use, psychosocial stressors, health behaviors, BMI, diabetes. |
| Tu et al., 2018 | To investigate the longitudinal association between social  participation and hypertension in China | China  (2011-13) | Longitudinal  China Health and Retirement Longitudinal Study | Middle-aged and older adults (45+ years)  (n = 5483) | Social participation (frequency of attending 5 activities in last month) | Incident hypertension | Among women, social participation once a week or more lowered the incidence of hypertension (IRR 0.80 [95% CI: 0.67, 0.95]).  Among men, no association was found | age, marital status, education, BMI, urban/rural, smoking, alcohol, SRH, depressive symptoms. |
| Wang et al, 2024 | To assess the relationship of social isolation and loneliness with hypertension risk among middle-aged and older adults in China | China (2011-2015) | Longitudinal  China Health and Retirement Longitudinal Study | Middle-aged and older adults (45+ years)  (n=3711) | Social isolation index at baseline | Odds of new-onset hypertension | People with high social isolation had 40% higher odds of hypertension onset (OR 1.40 [95%CI: 1.09,1.79]). Each unit increase in social isolation score was associated with 14% higher odds of hypertension onset (1.14 [1.04,1.26]) | Gender, age group, education, smoking, drinking, BMI, co-morbidities |
| ***Changes in exposure only (social tie/s)*** | | | | | | | | |
| Schwandt et al., 2010 | 1. To determine if staying married is associated with a lower prevalence of hypertension in midlife compared with staying never married, staying divorced, or staying widowed | United States  (1987/9 to 1996/8)  4 waves | Longitudinal  Athero-sclerosis Risk in Communities  (ARIC) | African-American adults  (46 – 64 years)    (n = 3425) | Change in marital status between visit 1 & visit 2 (3 levels): 1) Stayed married (reference)  2) Stayed non-married  3) Any change | Mortality;  new CHD; new diabetes; current hypertension | There was no association between staying non-married or any change and prevalent hypertension in either males or females. | Age, education, gender, BMI, cholesterol, physical activity, smoking. |
| Yang et al., 2013 | 1. To test the effects of social integration on metabolic functions over time  2. To examine population heterogeneity in the effects | United States  (1998-2006)  5 waves | Longitudinal  Health and Retirement Study  (HRS) | Adults  (50+ years)  (n = 4323) | Summary of number of social ties in 5 domains of activity (marital status, contact with parents, contact with children, with neighbors and volunteer) | Mean levels and trajectories of high blood pressure (and other MetS components) across 3 timepoints | No effect of changes in social integration on metabolic disorders over time | age, sex, race, education, household income, smoking status, drinking, exercise, BMI, depressive symptoms, number of chronic conditions, and cardiometabolic medications |
| Dupre et al., 2015 | To examine lifetime exposure to divorce and incidence of Acute Myocardial Infarction (AMI) | United states  (1992-2010) | Prospective cohort  Health and Retirement Study (HRS) | Ever-married adults aged 45 to 80 years  (n=15,827) | Stability and changes in marital status (continuously married (reference), divorced, widowed, or remarried) and the cumulative number of divorces (0, 1, or ≥2 divorces) | Hazard ratio (HR) of heart attack | Among women, AMI risks were significantly higher in women who had 1 divorce (HR 1.24; (95% CI: 1.01–1.55), ≥2 divorces (1.77 [1.30–2.41]), and among the remarried (1.35 [1.07–1.70]), compared to staying married.  Among men, higher risks were seen only with a history of ≥2 divorces (1.30 [1.02–1.66]) compared to staying married. Men who remarried had no significant risk for AMI | Age, cohort, race, region, education, employment, occupation, health insurance, lives alone, no children, depressive symptoms, smoking, alcohol use, vigorous exercise, BMI, hypertension, diabetes. |
| Chen et al., 2020 | To examined longitudinal data from three large cohorts of young, middle aged and older adults, with repeated measurements of religious-service attendance and multiple health and wellbeing outcomes. | United States | Prospective cohorts:  1. HRS, 6 waves  (2008-16)  2. Nurses’ Health Study II (NHSII)*  (2001-8/9/13)  3. Growing Up Today Study (GUTS)*  (2007-2010/13) | Youth (23 y), middle-aged (47 y) and older adults  (69 y)    (n = 12,549)  *GUTS and NHSII data have common households | Repeated attendance at religious service (social participation) | Hypertension Prevalence | Frequent attendance at religious service was not linked to hypertension, stroke or heart disease. Risks were decreased or increased depending on the cohort | age, gender, race/ethnicity, marital status, geographic region, employment status, nightshift work schedule, socioeconomic status, health insurance, attachment, childhood-abuse victimization, & previous religious-service attendance |

SD, standard deviation; SBP, systolic blood pressure; DBP, diastolic blood pressure; HRT, hormone replacement therapy; BMI, body mass index; y, years; CHD, coronary heart disease; MetS, Metabolic Syndrome; IRR, incidence rate ratio; OR, odds ratio; 95% CI, 95% confidence interval.

**Supplementary Table 4. Overview of included study characteristics and contextual factors by region.**

| **Region** | **Study** | **Population** | **Sample size &**  **Sex distribution** | **Follow-up period** | **Contextual factors** |
| --- | --- | --- | --- | --- | --- |
| Iran | Hosseinpour-Niazi et al., 2014 | Adolescents and adults (15-90 yrs.) | N = 5221  F: 61% | 9.6 years | Life Expectancy (2022):^1^ 75  Social Security:^2^ 59% (60+)  Divorce Rates:^3^ 200/1000  Marriage Rates:^3^ 7.5/1000  Marriage equality in women’s rights:^1*^ No |
| China | Wang 2005 | Young and middle-aged women (20-59 y) | N = 2189  F: 100% | 5-6 years | Life Expectancy (2022): 79  Social Security:^2^ 95% (60+)  Divorce Rates:^3^ 2.04/1000  Marriage Rates:^3^ 4.8/1000  Marriage equality in women’s rights:^1*^ Yes |
|  | Wang et al 2023 | National cohort of older adults | N=8782 | 4 years |  |
|  | Wang et al 2024 | Men aged 80 and above | N=2009  M: 100% | 4 years |  |
| USA | Kutob et al 2017 | Postmenopausal women (50-79 y) | N = 79,094  F: 100% | 5-6 years | Life Expectancy (2022): 77  Social Security:^2^ 97% (60+)  Divorce Rates:^3^ 2.4/1000  Marriage Rates:^3^ 6.2/1000  Marriage equality in women’s rights:^1*^ Yes |
|  | Wilcox et al 2003 | Postmenopausal women (50-79 y) | N = 38,483 | 3 years |  |

^1^ SOURCE: [Data for United States, China, Iran, Islamic Rep. | Data (worldbank.org)](https://data.worldbank.org/?locations=US-CN-IR)

^2^ SOURCE: Gomari et al (2020). A glimpse at Iran’s pension Funds, Saba Pension Strategies Institute (<https://saba-psi.ir/wp-content/uploads/2020/01/Iran’s-Pension-Funds-at-a-glance.pdf>); [www.gov.cn](http://www.gov.cn); US Social Security Administration statistics.

^3^ [www.statista.com](http://www.statista.com) and US Centre for Disease Control

* Marriage equality in women’s rights include 5 categories: 1) no legal requirements for married women to obey husbands; 2) ability of women to be ‘head of household’ same way as men; 3) specific legislation for domestic violence; 4) ability of women to obtain judgement of divorce same as men; and, 5) women have same rights to remarry as men.
